# Factors associated with depressive symptoms in patients with acute coronary syndrome undergoing percutaneous coronary intervention: A prospective cohort study

**DOI:** 10.1002/nop2.171

**Published:** 2018-06-28

**Authors:** Mana Doi, Hiroki Fukahori, Yumiko Oyama, Kumiko Morita

**Affiliations:** ^1^ Graduate School of Health Care Sciences Tokyo Medical and Dental University Tokyo Japan; ^2^ Nursing Course, School of Medicine Yokohama City University Yokohama Japan; ^3^ Faculty of Nursing and Medical Care Keio University Kanagawa Japan

**Keywords:** acute coronary syndrome, depression, percutaneous coronary intervention, nurses, nursing

## Abstract

**Aim:**

To identify the association between possible factors and depression among post‐percutaneous coronary intervention patients with acute coronary syndrome.

**Design:**

Prospective cohort study.

**Methods:**

Sixty‐eight post‐percutaneous coronary intervention patients with acute coronary syndrome were enrolled between January 2016 ‐ June 2017. The Hospital Anxiety and Depression Scale scores at 1–3 months after discharge were regressed onto uncertainty in illness and other clinical factors based on the Roy Adaptation Model.

**Results:**

Thirty‐six patients were included in the final analysis. Higher baseline depression scores, higher changes in uncertainty in illness and feeling annoyed by troublesome tasks after discharge were associated with higher depressive scores at 1 month after discharge. Careful observation and support of patients’ ineffective responses in self‐concept mode may be effective in preventing depression.

## 1 INTRODUCTION

Myocardial infarction is a leading cause of death and suffered by many people worldwide (GBD Disease & Injury Incidence & Prevalence Collaborators, 2017; White & Chew, 2017; World Health Organization, 2017). It is well known that patients with myocardial infarction tend to have depression. The prevalence of depression among patients with myocardial infarction ranges from 16% to 25% (Denollet, Strik, Lousberg, & Honig, 2006; Thomas et al., 2011). Previous studies (Kala et al., 2016; Larsen, Vestergaard, Søndergaard, & Christensen, 2013) have indicated that patients are at an increased risk of depression after being diagnosed with myocardial infarction. Furthermore, 17% of patients diagnosed with both myocardial infarction and depression die within 6 months after the diagnosis of myocardial infarction (Frasure‐Smith, Lespérance, & Talajic, 1993; Hosseini, Ghaemian, Mehdizadeh, & Ashraf, 2014). Therefore, preventing and treating depression among patients with myocardial infarction are important.

In undertaking the prevention and treatment of depression, we have to consider procedures for the treatment of myocardial infarction. This is because the invasiveness and associated length of stay differ for different procedures. One of the main procedures used in the management of myocardial infarction is percutaneous coronary intervention (PCI). It is characterized by minimal invasion and short length of stay, which is different from other procedures. The number of centers capable of performing PCI has been increasing in Western countries (Cook, Walker, Hügli, Togni, & Meier, 2007; Langabeer et al., 2013) and the number of patients for whom PCI is suitable has been increasing worldwide. In Japan, PCI is recommended as a primary treatment for patients with acute myocardial infarction (The Japanese Circulation Society, 2013). Approximately 80% of patients diagnosed with myocardial infarction are treated by PCI after being diagnosed with acute coronary syndrome (ACS) (Kasanuki et al., 2005; The Japanese Circulation Society, 2013), which represents a clinical subset (Anderson et al., 2013). Given the increasing number of patients for whom PCI is suited globally, there is the need to discuss the prevention and treatment of depression among patients with ACS who have undergone PCI in consideration of the level of invasion and short length of stay.

### 1.1 Background

Many myocardial infarction patients suffer from depression and require prevention and treatment. However, reports on the treatment of depression in the context of cardiovascular disease are scarce and it has been suggested that cardiac patients are untreated (Dobbels et al., 2002). This may increase the prevalence and severity of depression among patients with ACS who have undergone PCI. Previous studies have reported on the risk of depression in patients with ACS who have undergone PCI. An observational study (Gu, Zhou, Zhang, & Cui, 2016) revealed that the prevalence of depression in patients who underwent PCI increased after PCI. Furthermore, patients who underwent PCI reported that they had concerns regarding their future health (Higgins, Dunn, & Theobald, 2000). Additionally, a qualitative study (Daly et al., 2000) clarified that patients with acute myocardial infarction, including those treated with PCI, experienced fear of recurrence after discharge. Therefore, some patients with ACS who underwent PCI would experience depression and deteriorated mental health, including fear of recurrence.

To overcome this underserved state of depression, there is the need to evaluate the causative factors of depression to discuss their prevention and treatment. A review of depressive factors in myocardial infarction (Doi‐Kanno & Fukahori, 2016) has reported several factors that cause depression in patients with myocardial infarction. However, little is known about depressive factors among patients who undergo PCI for ACS. Therefore, we investigated depressive factors among patients with ACS who have undergone PCI in developed countries with a high number of PCI procedures, including Japan. It is difficult for nurses to focus on the mental health of patients who undergo PCI during such short hospital stays, as well as cardiac treatment and it is essential to identify and provide suggestions regarding the prevention and treatment of depression among patients with ACS who undergo PCI.

### 1.2 Theoretical framework

**Figure 1:**
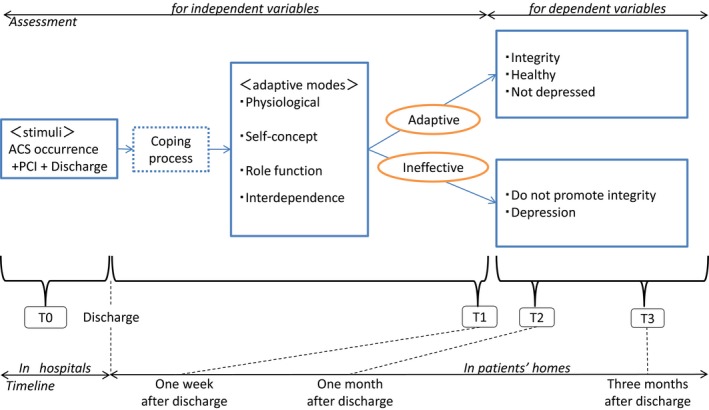
Research framework. ACS, acute coronary syndrome; PCI, percutaneous coronary intervention; T0, at hospitalization; T1, 1 week after discharge; T2, 1 month after discharge; T3, 3 months after discharge. T0–3: the timing of assessment

As shown in Figure 1, this study used the Roy Adaption Model (Roy & Andrews, 1991), which focuses on interactions with environmental change, as a conceptual framework. This conceptual framework was used to order possible depressive factors through the overview of the adaptation process in patients with ACS who have undergone PCI. We selected the Roy Adaption Model because the participants of this study experienced rapid environmental changes with the onset and treatment of their illness. According to this model, the person, as an adaptive system, is constantly interacting with a changing environment. Moreover, health is a reflection of this interaction. Thus, adaptive responses to the changing environment promote health, whereas ineffective responses do not. These responses were called behavior. They were divided into four categories (physiological, self‐concept, role function and interdependence) (Fitzpatrick & Whall, 2005; Roy & Andrews, 1991) and were examined as independent variables. We developed a research framework to examine predictors of depressive scores among patients with ACS who have undergone PCI (post‐PCI ACS patients). In this study, we aimed to identify the association between possible factors and depression among post‐PCI ACS patients.

## 2 THE STUDY

### 2.1 Design and participants

This was a prospective cohort study. The participants were patients diagnosed with ACS for the first time who underwent PCI at seven hospitals within the Tokyo metropolitan area between January 2016 and June 2017. The diagnosis of ACS was made by physicians. Patients were excluded if they: (a) were <20 or ≥80 years of age; (b) had undergone previous PCI; (c) had undergone previous coronary artery bypass grafting; (d) had difficulties in communication; (e) had experienced serious adverse effects from conditions other than complications related to ischemic heart disease, such as heart failure; (f) were unable to complete a questionnaire written in Japanese; (g) had not been aproved for participation by their physician; (h) had a diagnosis of depression or suspected depression after screening by the depression subscale of the Hospital Anxiety and Depression Scale (HADS‐D) at baseline (≥8 points) (Kitamura, 1993; Zigmond & Snaith, 1983); or (i) had a diagnosis of Takayasu's arteritis. Participants who met the inclusion criteria and responded at all follow‐up points were included in the analysis.

### 2.2 Method

#### 2.2.1 Procedure

Participants answered a questionnaire at hospitalization (T0) and at 1 week (T1), 1 month (T2) and 3 months (T3) after discharge. The assessment points of outcomes in this study (T2 and T3) were selected based on prior studies which examined the prevalence of depression in patients with myocardial infarction (Denollet et al., 2006; Kala et al., 2016; Larsen et al., 2013). The questionnaires for assessment at T0 and T1 were handed out at the hospitals and the completed questionnaires were mailed by the participants. The questionnaires for assessment at T2 and T3 were mailed only to participants who had completed the T1 questionnaire.

#### 2.2.2 Measures

##### Dependent variables

Our primary outcomes were the HADS‐D scores and the presence of depression according to the HADS‐D cutoff value at T2 and T3. In addition to HADS‐D scores, fear of recurrence and chest symptoms at T2 and T3 were also investigated as secondary outcomes of mental health (Daly et al., 2000).

The HADS is a 14‐item scale used to measure the levels of anxiety and depression of patients (Kitamura, 1993; Zigmond & Snaith, 1983). We used the Japanese version of the HADS scale which has good reliability and validity (Higashi et al., 1996). Each item is rated on a scale of 0–3, with seven items used to measure anxiety and seven items used to measure depression. For each subscale, the total score is 21, with higher scores indicating higher levels of anxiety or depression. We used the HADS‐D scores for dependent variables. A HADS‐D score of ≥8 indicates a suspected case and this was also used as the cutoff value (Kitamura, 1993; Zigmond & Snaith, 1983).

##### Independent and confounding variables

Both the independent and confounding variables for predicting HADS‐D scores were selected based on prior studies and the Roy Adaptation Model (Astin, Jones, & Thompson, 2005; Doi‐Kanno & Fukahori, 2016; Gravely‐Witte, Gucht, Heiser, Grace, & Elderen, 2007; Lauzon et al., 2003; Mortensen et al., 2007; Murphy et al., 2013; Naqvi et al., 2007; Norrman et al., 2004; Roy & Andrews, 1991; Schrader, Cheok, Hordacre, & Marker, 2006; Stafford, Jackson, & Berk, 2009). In this study, we estimated that the response (as independent variables) predict patients’ depression based on the Roy Adaptation Model.

According to Roy and Andrews (1991), an initial judgment as to whether the response is adaptive or ineffective plays an important role. To measure all independent variables, we assessed the changes in hospitalization and immediately after discharge responses. Post‐PCI ACS patients reported that they tended to have difficulty in comprehending physical changes soon after the procedure due to its minimally invasive nature and its performance soon after the onset of ACS (Gaw, 1992). Furthermore, considering poorer physical ability in hospitals and the increased physical activity after discharge (Taira, Nakamura, & Uchiumi, 2010), post‐PCI ACS patients will perceive a radical change in environment (i.e., stimuli in the Roy Adaptation Model) through the onset, PCI and discharge. In addition, assessment at T0 is the earliest point for post‐PCI ACS patients and it would reflect the status before the onset due to their difficulty in comprehension as stated above. Moreover, given the evaluation periods of all the assessment tools, we could perform the nearest assessment at T1 as an evaluation immediately after discharge. Then, we assessed the change in responses between T0 ‐ T1 and determined whether they were adaptive or ineffective. For categorical variables, we defined patients with symptoms or experiencing negative feelings at T1 as those with ineffective responses, whereas patients without symptoms or experiencing positive feelings at T1 were defined as those with adaptive responses. For continuous variables, we subtracted the scores at T0 from those at T1. For scores whose higher values indicated a better status, we designated changes in scores of <0 as ineffective and changes in scores of ≥0 as adaptive responses. For scores whose higher values indicated a poorer status, we subtracted the scores at T0 from those at T1 and designated changes in scores of >0 as ineffective and changes in scores of ≤0 as adaptive responses. We categorized a lack of change in scores as an adaptive response. This categorization was performed before the multivariate analysis.

##### Variables in physiological mode

“Physiological mode” is associated with the way a person responds physically to stimuli (Roy & Andrews, 1991). We included the presence of chest pain and angina in the physiological mode.

##### Variables in self‐concept mode

“Self‐concept mode” is defined as the composite of beliefs and feelings that a person holds about themselves at a given time (Roy & Andrews, 1991). HADS anxiety subscale scores, Universal Uncertainty in Illness Scale (UUIS) scores, feeling anxious about their cardiac condition after discharge, feeling annoyed by troublesome tasks after discharge, subjective physical status (according to the visual analog scale) and scores on the physical functioning subscale of the Medical Outcomes Study 36‐Item Short Form Health Survey (SF‐36) were included in the self‐concept mode.

We used the HADS anxiety subscale scores for independent variables and evaluated ineffective responses when the changes in scores between T0 ‐ T1 were >0.

The UUIS is a 26‐item Japanese scale developed by Nogawa (2012), based on the Mishel Uncertainty in Illness Scale. This scale is suitable for the Japanese population and can be administered regardless of the treatment setting (outpatient, community, or inpatient) with higher reliability and validity (Nogawa, 2012). Mishel (1988) defined uncertainty as a cognitive state and the inability to determine the meaning of illness‐related events. The total scores range from 26 ‐ 130, with higher scores indicating higher levels of uncertainty (Mishel, 1988; Nogawa, 2012). We evaluated ineffective responses when the changes in scores between T0 ‐ T1 were >0.

For evaluating subjective physical status, we used the visual analog scale. In this study, 100.0 mm horizontal lines were adapted (Polit & Beck, 2017). In the evaluation of subjective physical status, the anchors of the line ranged from 0 (most healthy) ‐ 100 (least healthy). We evaluated ineffective responses when the changes in scores between T0 ‐ T1 were >0.

We used the Japanese version of SF‐36, which has good reliability and validity (Fukuhara & Suzukamo, 2004). The SF‐36 comprises 8 subscales and is used to measure quality of life. Each subscale is given a score from 0 ‐ 100, with higher scores indicating a higher quality of life. We used only the physical functioning and social functioning subscales in this study. Furthermore, since the evaluation was undertaken at T0 and T1, we employed the acute version (where participants answered questions based on the preceding 1 week period) (Fukuhara & Suzukamo, 2004; Fukuhara, Bito, Green, Hsiao, & Kurokawa, 1998; Fukuhara, Ware, Kosinski, Wada, & Gandek, 1998). We evaluated ineffective responses when the changes in scores between T0 ‐ T1 were <0.

##### Variables in role function mode

“Role function mode” is defined in its expressive behavior as the feelings, attitudes, likes, or dislikes that a person has about a role or the performance of a role (Oda, 2009; Roy & Andrews, 1991). We included satisfaction with the performance of one's own role (the visual analog scale), General Self‐Efficacy Scale scores and changes in life perspective in the role function mode.

For evaluating the performance of one's own role, we used the visual analog scale. In the evaluation of the performance of one's own role, the anchors of the line were “no satisfaction” and “extreme satisfaction.” We evaluated ineffective responses when the changes in scores between T0 and T1 were <0.

The General Self‐Efficacy Scale is a 10‐item scale that measures self‐efficacy. We used the Japanese version of General Self‐Efficacy Scale which has good reliability and validity (Kawabata, Ikeno, Ugawa, Yuasa, & Kishi, 2011). The score on this scale reflects the strength of an individual's generalized self‐efficacy belief, with higher scores indicating a stronger generalized sense of self‐efficacy (Ito, Schwarzer, & Jerusalem, 2005). We evaluated ineffective responses when the changes in scores between T0 ‐ T1 were <0.

##### Variables in interdependence mode

“Interdependence mode” focuses on interactions related to the giving and receiving of love, respect and value (Roy & Andrews, 1991). The social functioning subscale of the SF‐36 was included in the interdependence mode.

##### Confounding variables

The confounding variables included age, sex, body mass index (BMI), a history of angina pectoris, the presence of residual ischemia after PCI, workplace considerations after discharge, the presence of cohabitants, return‐to‐work plans and the illness experience of patients or their families.

##### Participant characteristics

In addition to the abovementioned variables, we collected data regarding disease diagnosis, participation in cardiac rehabilitation programs, baseline HADS‐D scores, education level, marital status, smoking status and comorbidities.

### 2.3 Analysis

We performed a stepwise multiple regression analysis to identify the associated factors of depressive scores at T2 and T3. To confirm the correlations between the continuous independent variables before multiple regression analysis, we calculated Pearson's correlation coefficients for parametric scales and Spearman's rank correlation coefficients for non‐parametric scales. Furthermore, all of the characteristics associated with depressive scores in the univariate analysis (*p* < 0.25) at each follow‐up point were incorporated into the multiple regression analysis as potential confounding factors (Katz, 2006). All data were analyzed using SPSS Statistics 20.0 (IBM Corp., Armonk, NY, USA) and the significance level was set at α = 0.05.

### 2.4 Ethics

Informed consent was obtained from all participants who were enrolled this study at study centers. All institutional review boards approved this study prior to recruiting participants.

## 3 RESULTS

**Figure 2:**
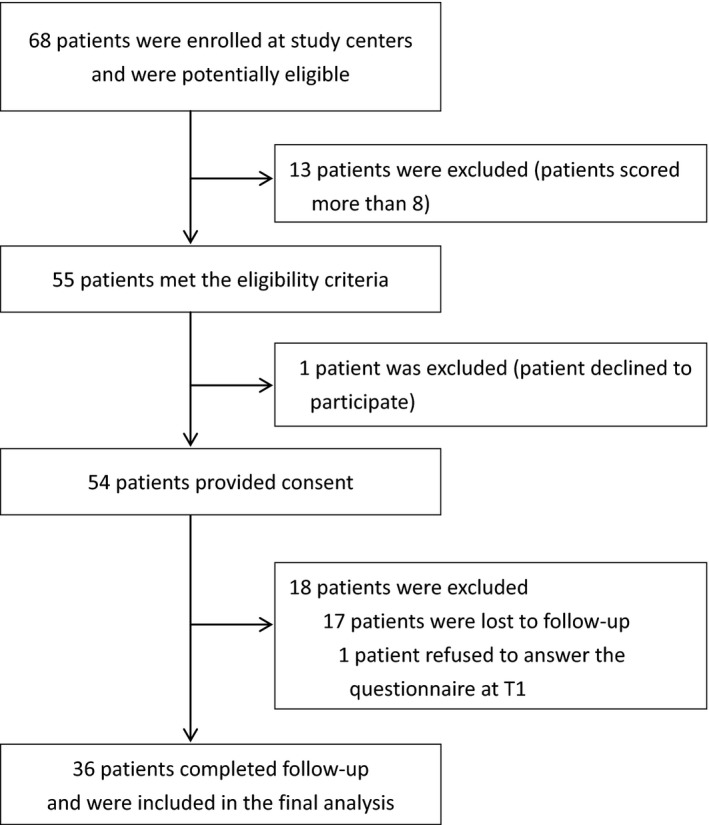
Flow chart of participant inclusion in this study. T1, 1 week after discharge

In this study, 36 of 68 participants were included and finally analyzed (response rate: 52.9%) (Figure 2). These 36 participants had no missing data. The mean age was 61.14 (standard deviation [*SD*] 11.13) years. The mean BMI was 24.93 (*SD* 3.72) kg/m^2^. Most patients were male (80.6%), had acute myocardial infarction (100.0%), did not participate in cardiac rehabilitation programs (75.0%), had completed high school (55.6%), were married (72.2%) and had a history of smoking (current or ex‐smokers) (63.9%). The characteristics of the patients are shown in Table 1.

**Table 1:** Demographic patient characteristics

Demographic variables	*N* = 36
Characteristics	
Age (years), mean (*SD*)	61.14 (11.13)
Male, %	80.6
Body mass index (kg/m^2^), mean (*SD*)	24.93 (3.72)
Acute myocardial infarction, %	100.0
Unstable angina pectoris, %	0.0
Participate in cardiac rehabilitation, %	25.0
HADS‐D scores at T0, mean (*SD*)	3.08 (2.17)
Education level	
Junior high school, %	13.9
High school, %	55.6
College or above, %	30.6
Married, %	72.2
Smoking status	
Current smoker, %	36.1
Ex‐smoker, %	27.8
Non‐smoker, %	36.1
Hypertention, %	41.7
Hyperlimidemia, %	11.1
Diabates, %	13.9
Angina pectoris, %	8.3
Residual ischemia after PCI	
Yes, %	13.9
Unknowm, %	47.2
Workplace considerations at post‐discharge	
Yes, %	19.4
Unknowm, %	13.9
Unemployed, %	50.0
Employer, %	5.6
Cohabitants, %	77.8
Must return to their work sooner, %	38.9
Illness experience of patients or their family, %	63.9
Independent factors: changes in T0 to T1 (adaptive response)	
Physiological mode	
Not having chest pain, %	91.7
Not having angina, %	97.2
Self‐concept mode	
Lower change in HADS anxiety subscale score, %^a^	36.1
Lower change in UUIS score, %^a^	27.8
Not feeling anxious regarding cardiac condition after discharge, %	30.6
Not feeling annoyed by troublesome tasks after discharge, %	80.6
Lower change in Subjective physical status(visual analogue scale), %^a^	66.7
Higher change in physical functioning score, %	83.3
Role function mode	
Higher change in satisfaction with the performance of one's own role (visual analogue scale）, %	55.6
Higher change in General Self‐Efficacy Scale score, %	44.4
Changes in life perspective, %	91.7
Interdependence mode	
Higher change in social functioning score, %	80.6

*Note*. *SD*, standard deviation; HADS‐D, the depression subscale of the Hospital Anxiety and Depression Scale; T0, at hospitalization; PCI, percutaneous coronary intervention; T1, 1 week after discharge; UUIS, Universal Uncertainty in Illness Scale.

a Higher scores indicate a poorer status.

Table 2 shows the data regarding HADS‐D scores, the presence of depression according to the HADS‐D cutoff value, fear of recurrence and fear of chest symptoms at T2 and T3. The mean HADS‐D scores at T2 and T3 were 2.69 (95%CI, 1.89–3.50) and 3.06 (95%CI, 2.17–3.94), respectively. Two patients with suspected depression were newly detected at T3 (5.6%). Most patients experienced fear of recurrence (T2, 66.7%; T3, 72.2%) and fear of chest symptoms at both follow‐up points (T2, 69.4%; T3, 72.2%).

**Table 2:** Outcomes at T2 and T3 *N* = 36

Dependent variables	Mean/proportion (95%CI)	
	T2	T3
HADS‐D		
Score	2.69 (1.89–3.50)	3.06 (2.17–3.94)
Cutoff (%)	0.0 (0.0– 9.7)	5.6 (0.7–18.7)
Fear		
of recurrence (%)	66.7 (51.3–82.1)	72.2 (57.6–86.9)
of chest symptoms (%)	69.4 (54.4–84.5)	72.2 (57.6–86.9)

*Note*. CI, confidence interval; T2, 1 month after discharge; T3, 3 months after discharge; HADS‐D, the depression subscale of the Hospital Anxiety and Depression Scale; *SD*, standard deviation.

Multiple regression analysis investigated the relative effects of factors on depressive scores at T2 and T3. In the multiple regression analysis, there were no strong correlations among independent variables (0.8 < |r|). Furthermore, we incorporated all other variables associated with each HADS‐D score at T2 and T3 (*p* < 0.25) into models as potential confounding factors.

In the analysis at T2, we included baseline HADS‐D scores, marital status, smoking status and diabetes as additional confounding factors. Table 3 shows the results of the multiple regression analysis of depressive scores at T2. Baseline depressive scores, changes in UUIS scores and feeling annoyed by troublesome tasks after discharge were associated with depressive scores at T2 (constant: 7.43 [*p* = 0.001]; baseline depressive scores: effect, 0.50 [*p* = 0.002]; changes in UUIS scores: effect, −1.65 [*p* = 0.016]; and feeling annoyed by troublesome tasks after discharge: effect, −1.87 [*p* = 0.024]).

**Table 3:** Multiple regression analysis of HADS‐D score at T2 *N* = 36

Variables	B (95%CI)	SE	β	*p* value
Baseline depression scores	0.50 (0.21–0.79)	0.14	0.46	0.002
Lower change in UUIS scores^a^ (*Changes in UUIS scores*)	−1.65 (−2.99 to −0.32)	0.65	−0.32	0.016
Not feeling annoyed by troublesome tasks after discharge^b^ (*Feeling annoyed by troublesome tasks after discharge*)	−1.87 (−3.48 to −0.27)	0.79	−0.32	0.024
R^2^	0.50
Adjusted R^2^	0.46
*F* value	10.78^c^

*Note*. B: regression coefficient; β: standardized regression coefficient.

Variance inflation factor (min‐max): 1.00–1.15.

Residual normality (*p* = 0.30), Durbin‐Watson ratio: 2.39.

Name of variables in Italic.

HADS‐D, the depression subscale of the Hospital Anxiety and Depression Scale; T2, 1 month after discharge; CI, confidence interval; SE, standard error; UUIS, Universal Uncertainty in Illness Scale.

a Lower change in UUIS (adaptive response), 1; Higher change in UUIS (ineffective response), 0.

b Not feeling annoyed by troublesome tasks after discharge (adaptive response), 1; feeling annoyed by troublesome tasks after discharge (ineffective response), 0.

c *p* < 0.001.

Baseline HADS‐D scores, education level, smoking status, hypertension and diabetes were included as additional confounding factors in the multivariate analysis at T3. A multiple regression model was not built for the analysis at T3 because of the violation of a residual assumption.

## 4 DISCUSSION

In this prospective cohort study, 5.6% of post‐PCI ACS patients had suspected depression at T3 and there was no detection of newly depressed patients at T2. Meanwhile, this study also identified that about 70% of post‐PCI ACS patients experienced fear of recurrence and chest symptoms at T2 and T3. Hence, it is suspected that over half of post‐PCI ACS patients have some problems which would lead to the deterioration of their mental health. Furthermore, we cannot rule out the sample size, higher baseline depression scores, higher changes in UUIS scores and feeling annoyed by troublesome tasks after discharge were associated with higher depressive scores at T2.

Both changes in UUIS scores and feeling annoyed by troublesome tasks after discharge were defined as self‐concept modes of the Roy Adaptation Model in this study and identified as depressive factors (Roy & Andrews, 1991). Among post‐PCI ACS patients, ineffective responses in self‐concept mode may cause deterioration in mental health. The basic need of the self‐concept mode is psychic integrity (Roy & Andrews, 1991). Therefore, patients who did not adapt their psychic perspective would experience depression after discharge.

This study revealed that higher changes in UUIS scores were associated with higher depressive scores at T2. Several previous studies (Ahn, Lee, Chu, & Sohn, 2017; Giammanco & Gitto, 2016; Hoth et al., 2013), which examined patients with chronic conditions, similarly reported an association between uncertainty in illness and depression. Uncertainty in illness is indicated as a psychological stressor and is related to poor mental health (Mishel, 2003; Neville, 2003). Hence, nurses should reduce the uncertainty of post‐PCI ACS patients to prevent higher depressive scores. It has been suggested that providing information and explanation regarding treatment and medications to patients is effective in reducing uncertainty (Mishel, 2003). Encouraging communication with patients who have successfully managed their uncertainty is also considered an effective method (Mishel, 2003). Therefore, assessing changes in uncertainty in illness to identify high‐risk groups for depression among post‐PCI ACS patients and providing educational intervention from nurses and patient peer support for high‐risk patients would be beneficial.

Feeling annoyed by troublesome tasks after discharge was considered in the context of the workplace. We interpreted this result as some post‐PCI ACS patients could not cope with their tasks as well as they did before the onset of ACS because they had returned to the workplace without adjusting their work to their changed physical functioning (Gaw, 1992) and this would impose some psychological burden. Therefore, some adjustments in the patient's work are needed and there is a need for nurses to advise them in this regard. We estimated that post‐PCI ACS patients were depressed when they could not adjust their work and cope with their tasks as well as expected, resulting in psychological strain. With regards to feeling annoyed by troublesome tasks after discharge, it is suggested that checking the status of recovery at work and whether there are changes in their feeling about tasks by nurses, as well as, advising adjustments in the patient's work will reduce the risk of depression after discharge (T2).

Baseline depressive scores were also identified as depressive factors at T2. Both baseline depressive scores and a history of depression were associated with depression during follow‐up in previous studies of myocardial infarction (Schrader et al., 2006; Stafford et al., 2009). It is suggested that patients who are depressed at baseline become more depressed after discharge regardless of the procedure.

In a clinical setting, healthcare providers should be aware of the risk of deteriorated mental health status among post‐PCI ACS patients after discharge. This is because our results revealed that post‐PCI ACS patients required monitoring and treatment of mental health issues regardless of the minimally‐invasive procedure and short length of stay. To prevent higher depressive scores at 1 month after discharge, the identification of patients at a high‐risk of depression, including high baseline depressive scores and intervention is needed. According to Roy and Andrews (1991), the first step of nursing intervention involves gathering data concerning the patient's behavior and the current state of adaptation. Careful observation, both at hospitalization and in the first ambulatory setting, leads to the identification of high‐risk patients who could become depressed in the future. Also, for patients who plan to return to work, it is effective to recommend that they make adjustments to their work. Furthermore, the majority of patients with ACS experience some irreversible changes in physical functioning, which converts their conditions into chronic ones. Therefore, it is important for nurses to provide information regarding perspective, including treatment and medication, during the short period of hospitalization, to reduce uncertainty and prevent depression.

The strength of our study lies in the well‐organized conceptual framework guided by the Roy Adaptation Model (Roy & Andrews, 1991). The Roy Adaptation Model has been used in many studies on the adaptation of patients with various diseases. By using this theory, we could identify potential predictors among post‐PCI ACS patients. Also, the results of our study consisted of adaptation in self‐concept mode in the Roy Adaptation Model. Thus, using this theory provided effective assistance of implication of the association between patients’ response and their mental health. Moreover, the theory provides the idea of the research in a general systematic context (Polit & Beck, 2017), which was beneficial for organizing the purpose of the study. The research framework and results of our study should form the basis for clinical practice and future research regarding the mental health of post‐PCI ACS patients. Furthermore, to the best of our knowledge, this is one of the first studies to identify depressive factors prospectively in post‐PCI ACS patients. We identified changes in UUIS scores and feeling annoyed by troublesome tasks after discharge as newly discovered factors, as well as, baseline depression scores, which is an already established factor in patients with myocardial infarction (Doi‐Kanno & Fukahori, 2016; Schrader et al., 2006; Stafford et al., 2009).

There are several limitations in this study. First, the sample size was small. We might have only detected factors with relatively strong associative relationships and there might be other underlying ones. In addition, this study could not discuss the continuation of factors over 1 month after discharge because the multivariate regression model was not established at 3 months. Therefore, there is the need for a larger‐scale study. Moreover, study participants were enrolled within a limited region. It is also necessary to enroll participants from other countries where PCI is conducted. Furthermore, not all eligible participants were included in the analysis due to loss to follow‐up. Further larger‐scale studies are needed to confirm the findings of this study and establish a more robust predictive model of depression.

## 5 CONCLUSIONS

Some post‐PCI ACS patients experience depression and deteriorated mental health after discharge. Furthermore, higher baseline depression scores, higher changes in UUIS scores and feeling annoyed by troublesome tasks after discharge are associated with higher depressive scores at 1 month after discharge. Careful observation and support of patients’ ineffective responses in self‐concept mode may be effective in preventing depression. It is also suggested that nurses should treat and prevent the deterioration of mental health among post‐PCI ACS patients regardless of the minimally‐invasive nature of the procedure and short length of stay.

## CONFLICT OF INTEREST

There are no conflicts of interest to declare.

## AUTHOR CONTRIBUTIONS

All authors meet your journal's criteria of authorship.

MD, HF and YO: Design. MD: data collection. MD, HF, and YO: analysis. MD, HF, YO, and KM: manuscript preparation.

All authors have agreed on the final version and meet at least one of the following criteria (recommended by the International Committee of Medical Journal Editors [https://www.icmje.org/recommendations/]):

- substantial contributions to conception and design, acquisition of data, or analysis and interpretation of data;
- drafting the article or revising it critically for important intellectual content.
